# Enhancing User Experience of Eye-Controlled Systems: Design Recommendations on the Optimal Size, Distance and Shape of Interactive Components from the Perspective of Peripheral Vision

**DOI:** 10.3390/ijerph191710737

**Published:** 2022-08-29

**Authors:** Yafeng Niu, Jingze Tian, Zijian Han, Mengyuan Qu, Mu Tong, Wenjun Yang, Chengqi Xue

**Affiliations:** 1 School of Mechanical Engineering, Southeast University, Nanjing 211189, China; 2 National Key Laboratory of Science and Technology on Aircraft Control, Xi’an 710065, China

**Keywords:** eye-controlled interaction, position, size, shape

## Abstract

For special populations with motor impairments, eye-controlled interaction may be the only way for them to communicate with the outside world. Because of the dominance of vision in the motor mechanism, eye-controlled interaction has high usability and important research value. During eye-controlled interaction, the visual channel needs to receive information from the graphical user interface (GUI) and transmit the user’s eye-controlled instructions, which overburdens the visual channel and reduces the efficiency of eye-controlled interaction. This study presents an ergonomic experiment to study how to design interactive GUI components in an eye-controlled user interface. The experiments were conducted based on the shape, size, and distance (from the object to the center of the screen) of the visual interactive components. The experiment comprised three parts: (1) the pre-experiment determined the evaluation index and selected the icon material; (2) the formal experiment was a three-factor within-subjects experiment, which included a search task using participants’ peripheral vision; and (3) after the experiment, subjective evaluations were conducted using a questionnaire. The results showed that the shape, size, and distance of the interactive object significantly affected the reaction time, and the size factor significantly affected the movement time of the eye-controlled interaction. Finally, combined with the results of the subjective evaluation, we concluded that the recommended sizes of the interactive components were 2.889°, 3.389°, and 3.889°, and the recommended distances were 5.966° and 8.609°. Additionally, designers should utilize components with simple concrete shapes as much as possible to improve user recognition efficiency. Our study provides enlightening recommendations on how to design components in eye-controlled interactive interfaces, and has great guiding significance for building design standards of the eye-controlled systems.

## 1. Introduction

With the development of human-computer interaction technology and improvement in the accuracy of eye tracking systems, the research on eye movement is changing from ‘passive’ to ‘active’. Eye-controlled interaction means that users can complete the interactive behavior by moving only through their eyes and the computer. An eye tracker is usually used to collect information from eyes. Other methods use a search coil [1] or are based on electrooculography [2]. Corresponding to the three basic physiological movements of gaze, saccade, and following, eye-controlled interactions can be divided into fixation, eye gesture, and smooth pursuit [3,4]. The earliest eye-controlled interaction was mainly used in some electronic communication devices in the 1970s [5,6]. Eye-controlled interaction was regarded as a feasible solution to replace the mouse and keyboard to control a computer. It can enable special groups with movement disorders (especially patients with amyotrophic lateral sclerosis, ALS) to control the computer without touching it [7,8,9]. The most famous eye-controlled typing application, Dasher, can reach a typing speed of up to 17 words per minute (wpm) [10]. Other eye-controlled typing applications such as EyeWrite can reach 8 wpm [11], and these applications can greatly help people relying on eye-controlled typing. At present, eye-controlled interaction has been applied in more contexts, such as auxiliary manipulation tasks [12,13], improving game experience [14], gaze-based painting [15], and designing authentication methods [16]. The eyes usually reach the next object in the sequence before any sign of manipulative action, which indicates eye movements are planned in the motor pattern and lead each action [17]. Therefore, compared to other interaction methods in human–computer interaction (e.g., hand gesture and intelligent speech interaction), eye movements can be a rapid, effective, and implicit way to communicate. However, eye-controlled interaction also has unique problems.

A phenomenon called ‘Midas touch’ often occurs while interacting with eye movements, which greatly reduces the usability of eye-controlled applications [18]. In the existing literature, research methods for optimizing eye-controlled interaction are mainly divided into two categories: (1) designing optimal eye-controlled interaction moves; and (2) using graphical user interface (GUI) elements. Most previous studies are in the former category, focusing on reorganizing and encoding basic eye-controlled actions or designing new eye-controlled actions to increase the complexity of the interaction process [19,20,21]. However, an important problem exists when designing eye-controlled interactions. In most eye-controlled applications, users need to communicate with computers for a long time. Simple eye-controlled actions are beneficial for reducing the users’ memory cost but are easily confused with natural eye-controlled actions. Furthermore, complex eye-controlled actions create a certain memory burden for the users and increase their mental load. Comparatively, using GUI elements in the process of eye-controlled interaction is a more feasible method. However, there is a lack of research on designing GUI elements to assist eye-controlled interactions.

This study aimed to explore the influence of GUI elements on the performance of eye-controlled interaction through specific and quantified selection of task results, and to provide inspiring suggestions for the design of eye-controlled interfaces in the future. To this end, the following section introduces the process of eye-controlled interaction and GUI elements, peripheral vision in eye control, visual variables in the eye-controlled interface, and the research content of this paper.

### 1.1. The Process of Eye-Controlled Interaction and GUI Elements

GUI elements include windows, icons, menus, pointing devices (also called WIMPs), or other visual representations. These elements play an auxiliary role in eye-controlled interactive interfaces. Previous studies have shown that GUI elements in the interface greatly impact the process of performing eye-controlled operations. Jenke found it necessary to provide users with relevant visual information in the process of performing eye gesture operations, and the visual interface elements they designed can have a significant guiding effect on eye gesture interaction [22,23]. The number of visual objects on a computer interface significantly impacts the efficiency with which users control their eyes [24]. In terms of eye-controlled applications, many designers have designed novel eye-controlled typing keyboards using WIMP elements to provide a better experience for users. For example, the eye-controlled typing application EyeWrite has four trigger areas hidden in four corners of the screen as pointing devices (pointers), and users can execute different interactive commands by looking at pointers in different sequences [25]. The Eye-S is a similar eye-based application with nine hidden rectangular trigger areas [11]. Urbina and Huckauf designed a typing application called pEYEwrite based on pie-shaped menus that can input letters by eye-controlled clicking on two pie-shaped menus respectively [26]. Similarly, the eye-controlled typing app designed by Bee and André uses a circular menu in which the user performs two single gaze gestures to input a letter [27]. Other researchers have used auxiliary patterns composed of visual representations, such as points, lines, and planes as feedforward to guide eye-controlled actions [28,29,30].

**Figure 1:**
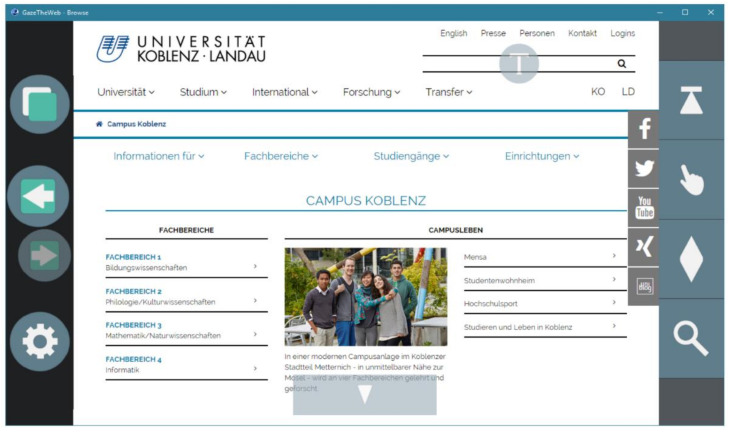
An eye-controlled browser based on eyeGUI [33].

In the research and design of eye-controlled interactions aided by GUI elements, the most important application approach is to design an eye-control browser. Eye-controlled browsers, such as GazeTheWeb [31] and WeyeB [32], use GUI elements that effectively guide users to perform eye gestures and contribute to improving interactive accuracy. In addition, Menges designed an eye-controlled user interface framework, eyeGUI [33], as shown in Figure 1. It supports users in using the daily browser in an eye-controlled interactive manner.

For example, when using an eye-controlled browser, the user first reads the content from the interface and then conveys interactive instructions through eye movements. The visual channel simultaneously receives information and transmits eye-controlled instructions. Visual fatigue and reduced efficiency of users performing eye-controlled interactions is caused when inappropriate GUI elements are used in the interface. Therefore, in-depth research must be conducted on how to design GUI elements for eye-controlled interactions.

### 1.2. Peripheral Vision in Eye-Controlled Interaction

When performing eye-controlled interaction with a GUI, the visual channel receives a large amount of information, including content information from web pages and interactive information from applications. Our visual processing ability is limited and selective when a large amount of visual information is received. This selection mechanism is called selective attention [34]. Selective attention enables users to focus on the useful information. Pre-attention occurs before selective attention and these two stages occur sequentially [35,36]. Pre-attention is very important for completing interactive behavior through eye control. For example, after reading the content for a period of time, people must first search for interactive components through pre-attention and then trigger them through eye movement. If the interface contains unreasonable interactive components (which are too small or too large), redundant eye movements can easily lead to eye strain and fatigue.

Human vision involves two mechanisms: focal and peripheral vision. Information obtained from attention and preattention is related to focal and peripheral vision, respectively [37]. In focal vision, the field of view is small, but the image has high resolution, which is the opposite in peripheral vision. High-resolution clear images from focal vision are advantageous for recognizing objects, whereas low-resolution images from peripheral vision can provide suitable indications of eye movements [38]. Paillard and Amblard argue that information in focal and peripheral vision is processed by two semi-independent visual systems, each of which plays a specific role in goal-directed movement [39]. Further research suggests that low spatial frequency (LSF) information in peripheral vision is helpful for predicting the direction of subsequent movements [40] and is also beneficial for processing high spatial frequency (HSF) information in focal vision [41]. This makes the study of peripheral vision useful in some ergonomic studies, such as in the design of head-mounted displays in aircrafts [42] and driving tasks [43]. However, there have been no ergonomic studies related to peripheral vision and eye-controlled interactions.

In this study, we designed a visual search task that combines eye-controlled interactions with peripheral vision. In the experiment, the participants had to use their peripheral vision to search for objects. Based on visual search performance through peripheral vision, this study aimed to gain insight into the design of useful eye-controlled GUI components.

### 1.3. Visual Variables in Eye-Controlled Interface

While using eye-controlled applications, users usually search for GUI components first, and then trigger them. This process is affected by several factors from the interface. Bertin defined position, size, shape, lightness, color, and texture as the six most important factors, also called as ‘visual variables’ [44]. These factors are closely related to the visual representation of an eye-controlled system. For example, color can significantly affect visual search efficiency [45], but Weelden et al. argued that color and texture would lead to a high cognitive processing load [46]. Therefore, we mainly studied shape, position, and size from a cognitive perspective and eye-controlled interactive component design. GUI can vividly convey metaphorical information and achieve a user-centered interactive experience [47,48]. Furthermore, shape factors play an important role in object perception [49,50] and are considered one of the main tools for object classification [51,52]. After accepting the visual information of the interface, the user first identifies the control by the form factor and subsequently associates the corresponding function of the control. According to the visual metaphor theory, one image can indicate another object by comparing images [53,54]. Metaphor classification of visual objects is closely related to shape factors [52]; therefore, we focus on the impact of visual metaphors of component shapes on interaction processes. Position and size represent the spatial characteristics of the interactive objects. The usability of eye-controlled components depends on their spatial characteristics. For example, compared to traditional browsers, eye-controlled browsers have larger interactive components to adapt to low-precision eye tracking systems [55]. From the human–computer interaction perspective, the spatial characteristics of GUI components have a significant impact on the user’s operational performance. This can be explained by Fitts’ law, which was originally a behavioral model for evaluating touch-and-click tasks [56], but Ware and Mikaelian found that eye-tracking input also obeys the same law [57]. Therefore, this study focused on the shape, position, and size of eye-controlled GUI components, among which the position factor can be expressed in the form of polar coordinates (direction and distance).

### 1.4. The Research Content of This Paper

This study investigated the effects of shape, position, and size on the performance of eye-controlled interaction through ergonomic experiments and drew conclusions about eye-controlled interactive component design. We designed an experimental paradigm to simulate visual-search tasks through eye-controlled interactions. The paradigm was divided into two parts: visual search and eye-controlled triggering. To draw a conclusion that is more aligned with the actual application situation of eye-controlled interaction, the two processes relied on peripheral vision and theories related to Fitts’ law. In the experiment, we chose some icons to simulate the visual search objects, taking the size, position (the distance from the icon to the center of the screen), and shape of the icons as independent variables. Additionally, we used the correct rate, reaction time (corresponding to the visual-searching process), and movement time (corresponding to the eye-controlled-triggering process) as dependent variables. We designed a within-subjects experiment to determine the influence of shape, position, and size of eye-controlled GUI components on the visual search task and obtained the optimal value and ideal combination of each factor.

The eye-controlled search task was divided into two processes in the sequence of time: visual-searching process (Stage 1) and eye-controlled-triggering process (Stage 2). We make the following assumptions based on relevant research:

**H1.**  *In Stage 1, the focal vision is clear but the peripheral vision is blurred, which is not conducive to the rapid identification of the target. It can be inferred that the size and distance of eye-controlled GUI components will significantly impact the performance of the visual-searching process.*

**H2.**  *In Stage 2, the predictive model of human movement used in ergonomics, i.e., Fitts’ Law, argued that the size and distance to target in a selection task would affect the performance of the act of pointing* [58,59,60]. *Like the pointing action in Fitts’ law, eye-controlled interaction also accomplishes the task through a single channel. Therefore, it can be assumed that the size and distance to components will significantly affect the eye-controlled-triggering process.*

**H3.**  *In Stage 1, the shape factor mainly affects the classification task of visual objects* [49,50,52]. *Stage 2 is performed after Stage 1 occurs. Therefore, the shape of the interactive objects will not significantly affect the eye-controlled-triggering process (Stage1).*

## 2. Experiment Method

### 2.1. Pre-Experiment: Screening Icons Based on Shape Classification Criteria

#### 2.1.1. Preparation

**Figure 2:**
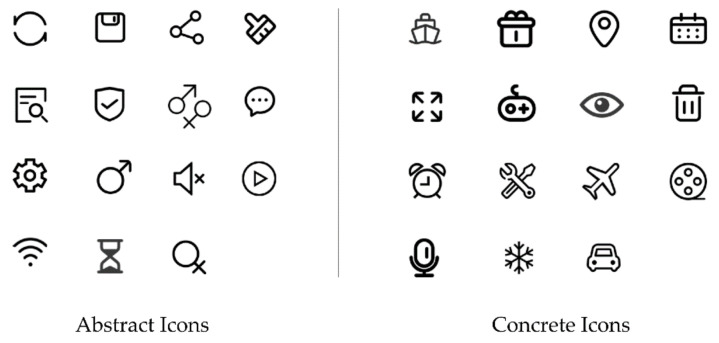
Icons used in the experiment.

The pre-experiment aimed to screen the icons for the formal experiment and determine the standard for the qualitative division of the shape variable of the icons. Combined with the discussion on shape and visual metaphors in Section 1.3, we divided icons into two types: concrete and abstract shapes. A concrete shape refers to an icon whose metaphorical object is similar to the image description, whereas an abstract shape refers to an icon whose metaphorical object is far from the image description. We selected 30 icons seen in daily life as experimental materials, divided into 15 abstract and concrete shapes each, as shown in Figure 2. All icons were flattened and processed using the Adobe Photoshop 2020 (Adobe Inc., San Jose, CA, USA). The content of the icons was related to an interactive interface, information communication, and transportation.

#### 2.1.2. Participants and Task Design

The pre-experiment included 14 participants (7 males and 7 females). Their ages ranged from 21 to 24, with an average age of 21.86 and an age variance of 0.901. They were all undergraduate or graduate students from School of Mechanical Engineering, Southeast University. All participants were physically and psychologically healthy.

The method of screening for icons was closely related to human cognitive processing. Snodgrass proposed four standardized indices related to memory and cognitive processing, namely, visual complexity, familiarity, image agreement, and name agreement [61], in which image agreement is consistent with our division criteria for icon shape. To prevent the other three variables from affecting the experiment, we used all four variables as indices for evaluating icons.

**Figure 3:**
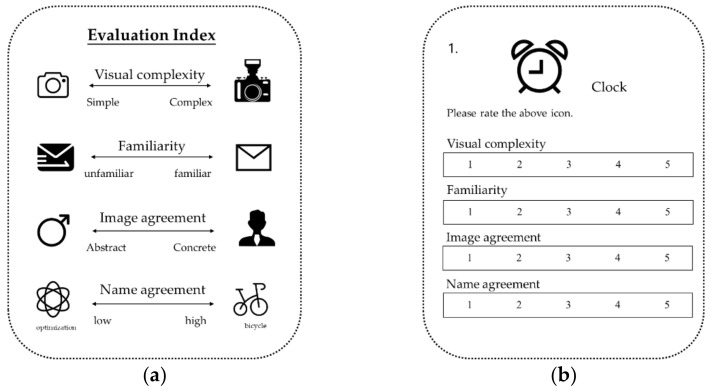
(**a**) Experimental evaluation index: visual complexity, familiarity, image agreement, and name agreement; (**b**) evaluation scale.

The experiment was conducted using a 5-point Likert scale. The scale is shown in Figure 3b. The participants rated each of the 30 icons according to the four indices. The four evaluation indices of the scale are shown in Figure 3a, and their meanings are as follows:

- Visual complexity represents the simplicity or complexity of the visual features of the icons. When there are many visual features, the amount of information received by the observer will increase.
- Familiarity indicates whether people are familiar with the icons. The more often an icon appears in daily life, the more familiar it is.
- Image agreement indicates how closely each icon resembles subjects’ mental image of the object, which can also be defined as the consistency between icon and the imagination. The icons with high consistency are concrete, whereas those with low consistency are abstract.
- Name agreement indicates the consistency between the name of the icon and its actual meaning. If the subjects can accurately understand the meaning of the icon after recognizing its name, it indicates that the icon has a high name agreement.

#### 2.1.3. Results and Data Analysis

The descriptive statistics of the abstract and concrete icons are presented in Table 1. The mean visual complexity scores of the abstract and concrete icons were approximately 2.0, and the mean familiarity and name agreement scores were similar. The mean value of image agreement was 3.78 and 3.05 for concrete and abstract icons, respectively. This means that the participants in the pre-experiment were more likely to produce physical mapping after recognizing the concrete icons rather than abstract icons.

Based on the total rating of each icon, eight abstract icons and eight concrete icons were selected for the final experiment. The selected concrete icons were: ‘Car’, ‘Voice’, ‘Delete’, ‘Tool’, ‘Movie’, ‘Plane’, ‘Snowflake’. The selected abstract icons were: ‘Mute’, ‘Chat’, ‘Time’, ‘Settings ‘, ‘Network’, ‘Play’, ‘Male’, ‘Female’.

ANOVA was conducted on the four standardized indices, and the results showed that there was no significant difference between the visual complexity, familiarity, and name agreement of the abstract and concrete icons (*p* > 0.05). There was a significant difference between the image agreement of icons in the two groups (*p* < 0.05), indicating that the three indexes other than image agreement had less influence on the perception of icons in this experiment, which also indicated that the grouping of icons in this experiment was reasonable. After screening the icons as material through the pre-experiment, the formal experiment used these screened icons for further research.

### 2.2. Experiment

A total of 15 abstract and 15 concrete icons were selected in the pre-experiment, which were used to simulate the eye-controlled GUI components in the experiment. The experiment studied the effects of size, distance, and shape of the components on the performance of the visual search task through peripheral vision.

#### 2.2.1. Experiment Design

The experiment was a 2 × 3 × 5 within-subjects design. The independent variables were the size, shape, and the distance to the to-be-selected icon. The dependent variables were correct rate, reaction time, and movement time to measure the interactive performance in the task. The specific variables and levels are shown below:

1. Independent variables:
  - Icon shape: concrete shape and abstract shape.
  - Icon distances (distance from the center of the interface to the center of the icon): 243 px (5.966°), 351 px (8.609°), 459 px (11.243°).
  - Icon sizes (expressed as the side length of the rectangular base): 80 px (1.889°), 100 px (2.389°), 120 px (2.889°), 140 px (3.389°), 160 px (3.889°).
2. Dependent variable:
  - Correct rate: The ratio of the number of correct trials to the total number of trials for each combination of variable levels. The correct rate was used to measure the likelihood of user errors when performing a visual search task in an eye-controlled interface.
  - Reaction time: The time taken by the participant from receiving the stimulus to completion of the visual search task.
  - Movement time: The time taken by the participant to perform the eye-controlled triggering process on the search object.

There were 30 combinations of variable levels in the experiment (2 × 3 × 5 = 30), and four replications of each variable combination were performed, resulting in 120 trials (30 × 4 = 120). In each task, participants needed to complete saccades in different directions to complete eye-controlled triggers, and studies have suggested that the movement time of saccades is related to the direction [62]. To eliminate the effect of orientation on performance, the experiment was set up with four replicate experiments with each combination of independent variable levels. The four replicate experiments were identical, but the target to be selected would appear in any direction (any of the eight orientations) to exclude the effect of the direction of saccades. At the end of the experiment, the performance data of the four repetitions were averaged to obtain the final data for the analysis. The statistical test power of the experiment was calculated using G*power 3.1.9.7 (created by Franz Faul et al., Kiel, Germany [63]), where the total sample size was 20, the number of groups was 1, and the number of measurements was 30 (2 × Shape + 3 × Distance + 5 × Size). The effect size was f = 0.25, α = 0.05, and the test efficacy (power, 1 − β) was 0.9999 (>0.8), so the probability of committing a Type-II error was less than 0.01%.

**Figure 4:**
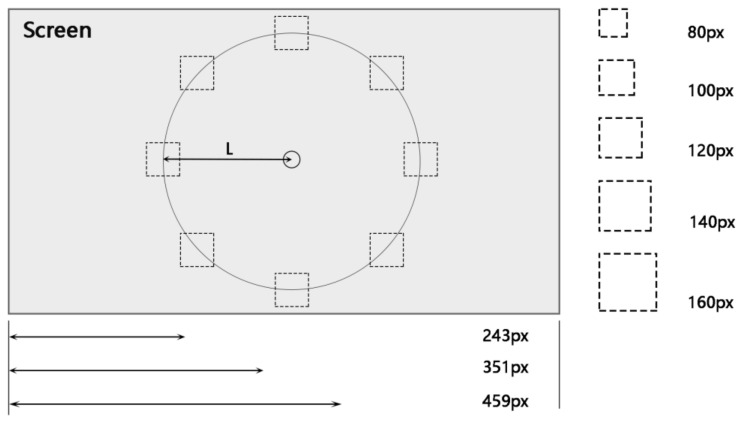
Diagram of experimental interface distribution, distance factor and size factor.

The interface distribution of this experiment is shown in Figure 4. A circular distribution interface was used, and the participants were required to perform visual search experiments on this experimental interface. The center ring is marked as the starting domain for the gaze, and the ending domain is set up with different icons, each with the same distance to the center of the circle. The surrounding area was set with eight icons to be selected, and the target randomly appeared at each of these eight positions with the same probability. In addition, the text cues used in the experiments were the names of the icons to be selected, and the cue font was Ariel, with a size of 33 px and a style of Normal. The diameter of the central marker was 60 px.

#### 2.2.2. Participants, Apparatus, and Experiment Environment

Twenty participants (10 men and 10 women) were invited to participate in this experiment. The participants’ ages ranged from 22 to 26, with a mean age of 22.7 and an age variance of 2.6. All the participants were undergraduate or graduate students from Southeast University with good physical and mental health and no history of psychiatric disorders. All the participants had corrected visual acuity ≥1.0 and no eye diseases.

Experiments were conducted using a Tobii Eye Tracker5 eye-tracking device with Windows 10 (64-bit) RS3 or higher. The device has a sampling frequency of 133 Hz and offers excellent responsiveness, accuracy, and reliability, enabling pilot tracking using the latest smart sensor technology. The monitor used was a Dell SE2419H_HX model LCD monitor, 24′, with an aspect ratio of 16:9, brightness of 250 cd/m^2^, original resolution of 1920 px × 1080 px, and a pixel pitch (actual distance between pixels) of 0.2745 mm. The eye-tracking device was fixed in the lower center of the monitor and connected to the laptop through the USB port, with the monitor placed 64 cm directly in front of the user’s eyes. The experimental platform was built by importing the Tobii SDK installation package via Unity 6.0 and compiling it in C#.

**Figure 5:**
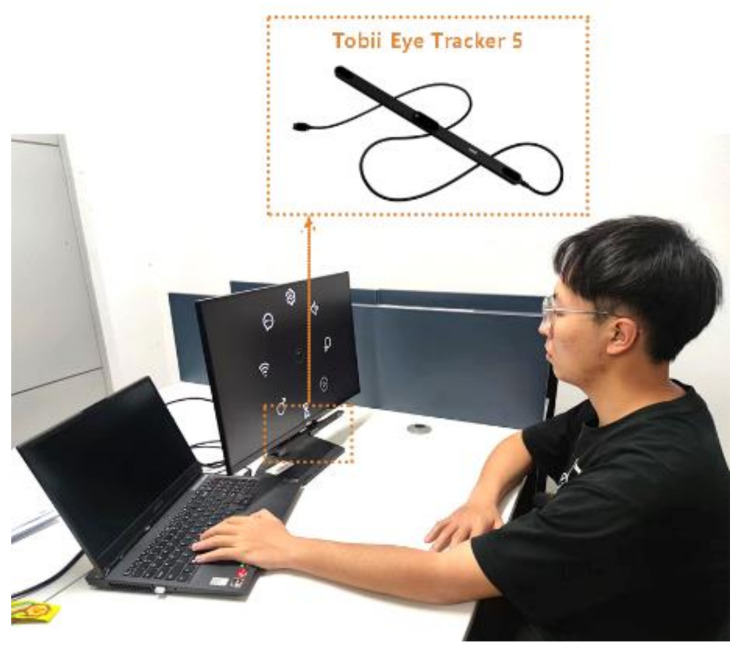
Experimental scenario.

The experiments were conducted in a laboratory with an area of about 30 m^2^, with natural light, good ventilation, and no noise interference. The experimental scenario is shown in Figure 5.

#### 2.2.3. Procedure

***Preparation***: First, the participants were asked to position themselves with 64 cm between their eyes and screen. The icons were then shown to the participants to ensure that they there was no ambiguity regarding the meaning of the icons. Because the Tobii eye-tracking device supports head movements, participants could move their head slightly during the experiment. Next, a pre-experimental calibration was performed. After a 7-point calibration with the Tobii Eye Tracker 5 (Stockholm, Sweden), a bubble pointer (also known as ‘Tobii Ghost’) appeared on the screen with gaze point feedback. After confirming that the participants gaze position and bubble pointer appeared in the same position, the calibration was successful; otherwise, the calibration session was repeated. After calibration, the experimental rules were explained to the participants, and the practice experiments began. These experiments were consistent with the formal experimental procedure; however, there were no data recording and no time limit.

**Figure 6:**
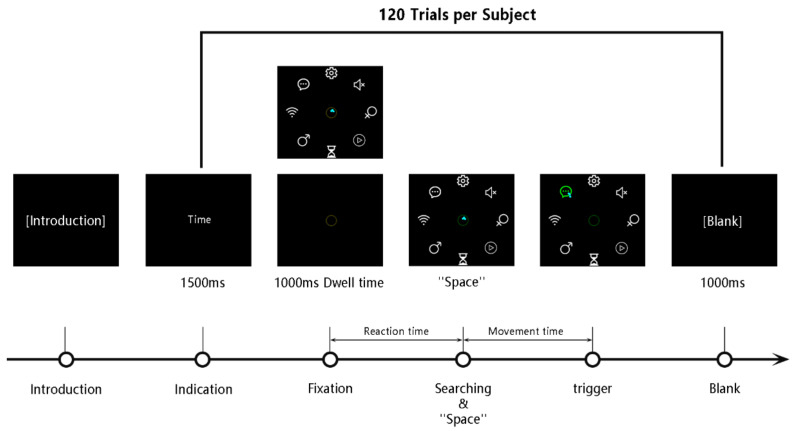
Flow chart of experiment.

***Single Trial***: The experimental procedure was the same for each trial in the formal experiment. This procedure is shown in Figure 6: (1) The name of the search target appeared in the center of the screen and disappeared after 1.5 s. (2) An annular marker appeared in the same position as the text. The participant needed to gaze at the annular marker to trigger it; the marker would become yellow during the gaze, and become green after 1000 ms. (3) Along with the trigger, eight to-be-selected icons would appear around the screen simultaneously. The participant was required to maintain their gaze and search for the task through peripheral vision. While finding the search target through peripheral vision, the participant was required to press the ‘Space’ button to indicate that the target had been found. (4) After pressing the ‘Space’ key, the subject could move the gaze point to the target icon to complete the eye-controlled-triggering process. (5) Finally, a blank screen was presented for 1 s to eliminate any visual residual image. The experiment was divided into four parts because of the fatigue that could be experienced by the participants. The participants could take a break with no time limit after every 30 trials and was informed of the overall progress of the experiment.

The three dependent variables of the experiment were reaction time, movement time, and correct rate. As shown in Figure 6, in the experiment, the reaction time is the time taken from the appearance of the icon until the participant presses the ‘Space’ key, which corresponds to the visual-searching process of the participant. The movement time is the time recorded from when the ‘Space’ key is pressed till completing the triggering, which corresponds to the eye-controlled-triggering process. Correct rate is the ratio of the number of correct selections to the total number of selections.

Eye movements are very fast, and humans usually look before the next action [64]. This subconscious habit would affect the experimental task; hence, the following protection mechanism was set up in the experiment. While the participant gazed at the central annular marker and searched for the target through peripheral vision, if the viewpoint moved away from the annular marker, the surrounding to-be-selected icons would disappear immediately. The participant was required to look at the central annular marker again to trigger it. The experimental trial was able to continue only when the visual search was completed (by pressing ‘Space’). Through this mechanism, the participants could complete the visual search task only through their peripheral vision, which guaranteed a real simulation of eye-controlled interaction.

#### 2.2.4. Parameter Set

Trigger time: Participants were required to gaze at the central annular marker for a certain amount of time; when the time spent gazing exceeded the time threshold, eight to-be-selected icons appeared. Many researchers have studied the time threshold of gaze interaction and found that the settings of gaze trigger time ranged from 200 to 1000 ms [65,66,67]. In this experiment, participants had to search for targets from the corners of their eyes. Therefore, to allow participants to produce complete peripheral vision in time after completing the gaze action, 1000 ms was chosen as the trigger time threshold for the central annular marker.

**Figure 7:**
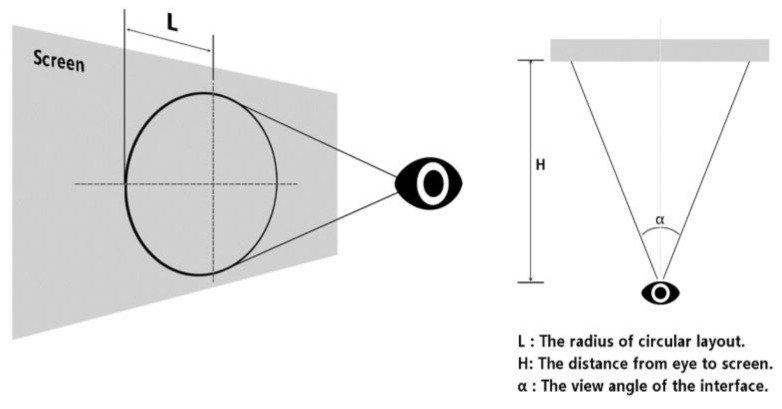
Schematic diagram of experimental target distance.

Icon size: Niu et al. studied the trigger accuracy and task completion time of visual search tasks in an eye-controlled interface with a grid interface layout [68]. They proposed a recommended size of 200 px (viewing angle of 2.889°) for the control. The icon-size determination method for this experiment is shown in Figure 7 and Equation (1).

(1) $$\alpha =2\times \tan^{-1}\frac{L}{H}$$

where α is the size of the viewing angle corresponding to the icon size, L is the side length of the square base of the icon, and H is the distance from the participant to the screen. Combined with the experimental equipment parameters in Section 2.2.2, we can calculate that the size corresponding to the angle of view of 2.889° in the experimental display is 120 px. The error of the eye-tracking instrument used in the experiment was 0.32° (approximately 22.5 px, which is approximated as 20 px), so the step size of the component was set to 20 px, which could result in two levels, larger and smaller than 120 px respectively. This resulted in five levels (80, 100, 120, 140, and 160 px), converted into angles of 1.889°, 2.389°, 2.889°, 3.389°, and 3.889°, respectively.

**Figure 8:**
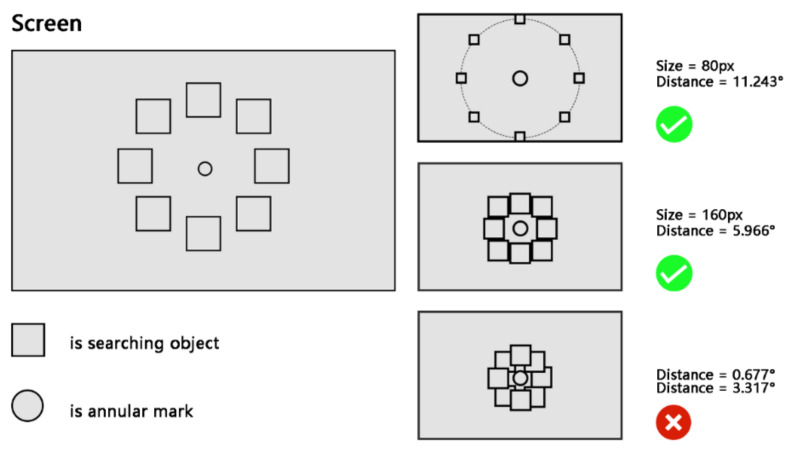
Extreme positions and overlap phenomena: ‘right’ indicates a normal condition that occurs in the experiment, and ‘cross’ indicates a condition that is not allowed to occur in the experiment.

Icon distance: The distance in the experiment was divided into three levels of 5.966°, 8.609°, and 11.243°, which were converted into pixels of 243, 351, and 459 px, respectively. The definition of distance in the experiment is the same as Fitts’ law [58,59,60], which refers to the distance from the center of the icon to the location of the central annular marker. The value of the distance factor considers the two extreme location distributions (closest and farthest positions from the central annular marker), as shown in Figure 8. Two distance values, 0.677° and 11.243°, were obtained based on the nearest and farthest extremes. The distance between the two extremes was divided into five levels of distance variables: 0.677°, 3.317°, 5.966°, 8.609°, and 11.243°. However, it was found that the icons of the to-be-selected targets in the interface would overlap when the distance was taken as 0.677° and 3.317°; therefore, these two levels were removed.

### 2.3. Questionnaire Evaluation

To verify the results of the experiments, we designed an immersive evaluation interface for a subjective evaluation. As in the formal experiment, the annular marker at the center of the interface was retained in the immersive evaluation platform, and changed to yellow when subjects gazed at it. Subjects could trigger the annular marker by gaze to recall the situation and experience of visual-searching process through peripheral vision, so that they could make real evaluation in the questionnaire.

**Figure 9:**
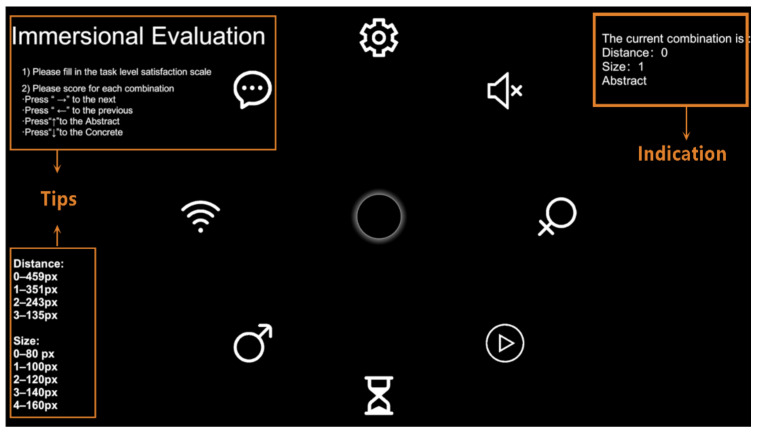
Immersive evaluation: The upper left is the operation method. The upper right is the current variable combination. The lower left is the number of distance and area variables. The current combination presented in the figure means the distance is 459 px (Marked as 0), the size is 100 px (Marked as 1), and abstract objects.

The evaluation interface was programmed using Unity3D and Visual Studio, as shown in Figure 9. All 20 participants (10 male and 10 female) who took part in the experiment underwent this immersive evaluation, which the participants underwent upon completion of the formal experiment to retain their memory and authentic experience of the experiment. The participants were asked to rate the combinations of variables presented in the evaluation interface. The rating index was the difficulty of the visual search task in the formal experiment, which was divided into a 5-point Likert scale: very easy, easy, fair, difficult, and very difficult. The central circular marker was the same as that in the formal experiment, and color feedback was presented when the viewpoint was inside the marker.

## 3. Results and Analysis

### 3.1. Experiment

A total of 2400 performance data and 600 subjective evaluation data were obtained, which were subjected to multi-factor ANOVA, simple effects analysis with significant interactions, and post hoc multiple comparisons for factors showing significant main effects.

#### 3.1.1. Correct Rate

**Figure 10:**
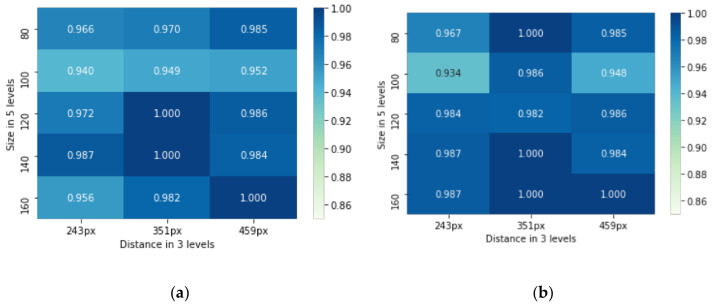
(**a**): Average correct rate of abstract icons; (**b**) average correct rate of concrete icons.

If the selected icon was not the prompted one, the trial was judged to be incorrect. The average correct rate for the 30 variables was 97.9%, indicating that all the participants in the experiment were able to complete the search task accurately. As shown in Figure 10a, the average correct rate of the abstract icons was 97.5%, and the abstract icon with a distance of 100 px and size of 243 px had the lowest correct rate of 94%. As shown in Figure 10b, the average correct rate of the concrete icons was 98.2%, and the concrete icon with a distance of 100 px and size of 243 px achieved the lowest correct rate of 93.4%. Therefore, from the descriptive statistics of the correct rate, the correct rate of the abstract icons was slightly lower than that of the concrete icons. According to the participants’ feedback after the experiment, this may be due to their misunderstanding of the meaning of the icons. To eliminate the ambiguity of the icons, we asked the participants to browse through all icons presented in the experiment before each experiment. However, there were still some who could not accurately understand the metaphors of the icons. For example, some participants easily confused the ‘male’ and ‘female’ icons in the abstract group.

A multi-factor ANOVA was implemented for the correct rate. The results showed a significant main effect of the size factor (F (4,1953) = 4.430, *p* = 0.001 < 0.05), and post hoc multiple comparisons showed that the area factor of 100 px was significantly different from that of 80 px (*p* = 0.009 < 0.05), 120 px (*p* = 0.002 < 0.05), 140 px (*p* = 0.000 < 0.05), and 160 px (*p* = 0.001 < 0.05), while the last four levels were not significantly different from each other. The main effect of the distance factor was not significant (F (2,1955) = 2.803, *p* = 0.061 > 0.05) and the main effect of the shape factor was also not significant (F (1,1956) =1.148, *p* = 0.284 > 0.05). It can be concluded that the distance and shape factors of the visualized object (icons) had no significant effect on the correct rate of the search task. Although the size factor had a significant influence on the correct rate, the correct rate did not show a monotonic change with the increasing size of the search object, and the correct rate of the search in an area of 100 px was significantly lower than that at other levels (levels larger or smaller than 100 px showed higher correct rates). Since the deviation of the average correct rate was not large (within 4% difference) and was higher than 93% overall, the impact of this phenomenon on the correctness of eye-controlled interaction is not significant.

#### 3.1.2. Reaction Time

**Figure 11:**
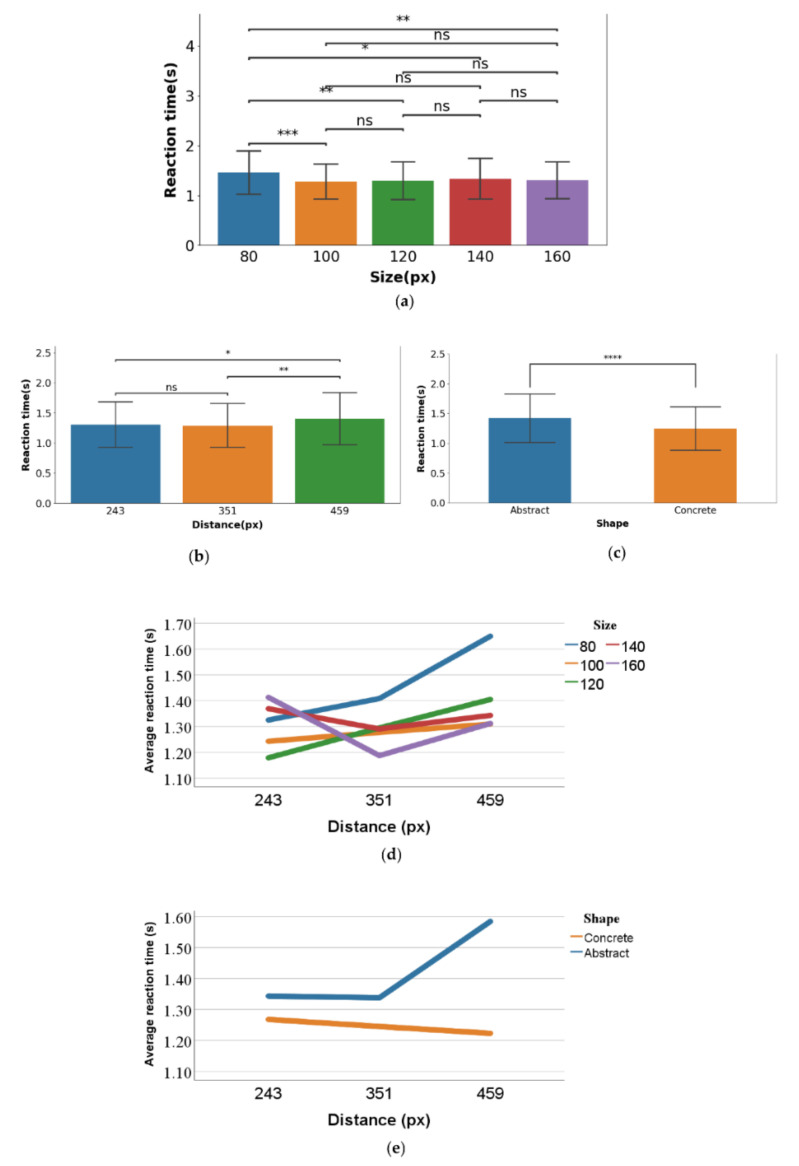
(**a**–**c**) Bar graphs of response times and post hoc multiple comparisons of main effect levels for the size, distance, and shape factors, respectively. The line between the levels indicates the results of the t-test at the level of the two variables, ‘ns’ indicates that no significant differences are presented between samples, ‘*’ indicates that significant differences are presented between samples, and ‘**, *** and ****’ indicates a higher significance. (**d**,**e**) show the simple effect analysis of interaction effect of distance × size and distance × shape, respectively.

The descriptive statistics for reaction time are shown in Table 2, and the results show that the average reaction times were 1.46, 1.27, 1.29, 1.33, and 1.30 s at the five size levels of 80, 100, 120, 140, and 160 px, respectively. The average reaction time for the distances 243, 351, and 459 px were 1.30, 1.29, and 1.40 s, respectively. The mean reaction times for the abstract and concrete icons were 1.42 and 1.25 s, respectively. The bar graphs of the reaction times at different size, distance, and shape levels are shown in Figure 11a–c, respectively.

Regarding the inferential statistics of the reaction time data, we first cleaned the data by excluding those with reaction times outside M ± 3SD (5.79% of the reaction time data) and then performed a normality test on the data. The results of the Shapiro–Wilk test showed that all the data under 30 combinations of variable levels were consistent with the normality assumption.

One-way Welch’s ANOVA was used to determine whether the size, distance, and shape factors had an impact on the reaction time. As shown in Table 2, the results showed that the main effect of the size factor (F (4,297.095) = 3.771, *p* = 0.005 < 0.05) was significant, and a post hoc test (Figure 11b) indicated that the size factor of 80 px was significantly different from that of 100, 120, 140, and 160 px, while the last four levels were not significantly different from each other. The main effect of the distance factor (F (2,395.822) 4.329, *p* = 0.014 < 0.05) was significant, and a post hoc test (Figure 11a) showed that the distance factor level of 459 px showed significant differences as compared to 243 and 351 px. The main effect of the shape factor (F (1,591.364) = 21.028, *p* = 0.000 < 0.05) was significant. Therefore, there were significant effects on all three factors: size, distance, and shape. It can be tentatively stated that eye-controlled GUI components with a larger size and longer distance from the center of the screen would lead to less reaction time for visual search. However, the reaction time did not change if the size was bigger than 80 px or the distance was longer than 351 px. As for the shape factor, the abstract shape would require a longer response time for the search task than the concrete shape.

A multi-factor ANOVA determined that the interaction effect of distance × size (F = 9.323, *p* = 0.000 < 0.05) and the interaction effect of distance × shape (F = 7.619, *p* = 0.001 < 0.05) was significant. According to a simple effect analysis of distance × size (Figure 11d), the average reaction times for sizes of 80, 100, and 120 px showed an increasing trend as the distance increased, but a trend of fall–rise appeared with sizes of 140 and 160 px. As shown in Figure 11e, a simple effect analysis of the interaction effect of distance × shape showed that the average reaction time of concrete-shaped objects did not change significantly with the increase in distance, and the average reaction time of abstract-shaped objects did not change significantly at a distance of 243 or 351 px but increased significantly at 459 px.

#### 3.1.3. Movement Time

**Figure 12:**
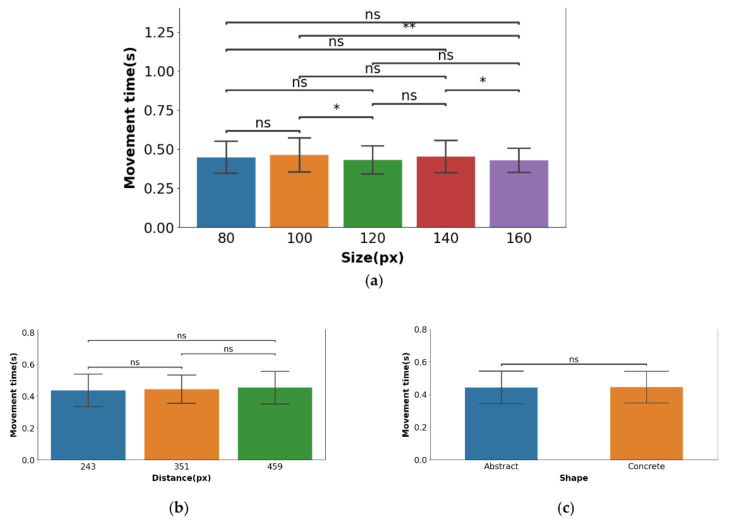
(**a**–**c**): Bar graphs of movement times and post hoc multiple comparisons of main effect levels for the size, distance, and shape variables, respectively. The line between levels indicates the results of the t-test at the level of the two variables, ‘ns’ indicates that no significant differences are presented between samples, ‘*’ indicates that significant differences are presented between samples, and ‘**’ indicates the higher significance.

The descriptive statistics are shown in Table 3, with average reaction times of 0.488, 0.463, 0.430, 0.452, and 0.428 s for sizes of 80, 100, 120, 140, and 160 px respectively. The average reaction times were 0.436, 0.444, and 0.453 s with the distances of 243, 351, and 459 px. The average reaction times for the abstract- and concrete-shaped objects were 0.444 and 0.445 s, respectively. The bar graphs of the reaction times at different distance, size, and shape levels are shown in Figure 12a–c, respectively.

Regarding the inferential statistics of the movement time data, we first performed data cleaning to remove data with movement times outside of M ± 3SD (13.08% of the response time data) and then performed normality tests on the data. The results of the Shapiro–Wilk test indicated that some of the data had significant deviations from a normal distribution (7 out of 30 variable levels), but the overall profile of the data was bell-shaped, so these data presented an approximate normal distribution, and ANOVA with an equal sample size was able to provide a valid measure [69].

One-way Welch’s ANOVA was used to determine whether the size, distance, and shape factors had an impact on movement time. As shown in Table 3, the results showed that the main effect of the size factor (F (4,292.339) = 2.866, *p* = 0.024 < 0.05) was significant, and a post hoc test (Figure 12a) showed that the size factor was significantly different between 100, 120, and 160 px, and the area factor was significantly different between 140 and 160 px. The main effect of the distance factor (F (2,390.868) = 1.349, *p* = 0.261 > 0.05) and shape factor (F (1,589.100) = 0.053, *p* = 0.818 > 0.05) was not significant. Therefore, it can be tentatively concluded that as the size of the search object increases, the movement time (i.e., the time to complete the saccades) of the visual search process will decrease.

### 3.2. Subjective Questionnaire

**Figure 13:**
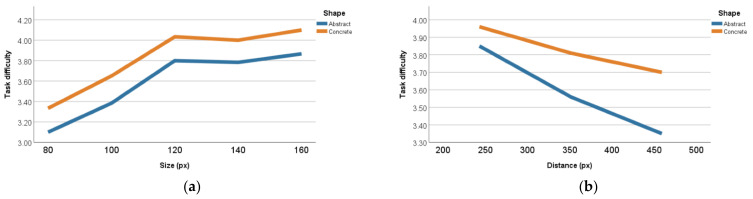
(**a**) The subjective evaluation analysis of size and shape; (**b**) the subjective evaluation analysis of distance and shape.

A total of 20 participants were asked to provide a subjective evaluation of the experiment on a scale of 1 to 5, with 1 corresponding to very difficult, and 5 to very easy. Descriptive statistics were derived for ratings of size, distance, and shape. As shown in Figure 13a, according to the results of the evaluation, the visual search task was more difficult when the size was 80 and 100 px, and easier when the size was 100, 120, and 160 px. As Figure 13b presents, the task was least difficult at a distance of 243 px and most difficult at a distance of 459 px; thus, the difficulty of the task increased as the distance increased. In addition, all the participants thought that the difficulty of the experiment was higher with abstract icons than with concrete icons, which means that concrete icons were more conducive to the performance of eye-controlled interaction.

## 4. General Discussion

This experiment aimed to determine the performance of the eye-controlled visual-search task under different levels of independent variables (size, distance, and shape). The visual search task in the experiment was divided into two processes: visual searching and eye-controlled triggering processes. The correct rate which was used to measure the feasibility of the experiment, and showed that all participants could complete the task accurately.

First, the most significant effect on reaction time showed that the reaction time of the visual search task increased as the size of eye-controlled GUI components decreased and as they moved further from the center of the interface. This result supports Hypothesis 1 (**H1**). Studies have shown that the reaction time for search tasks depends on the number of eye movements and the duration of a single gaze [70,71], and stated that the duration of a single gaze increases with the difficulty of the peripheral visual search task [72]. Thus, an increase in the difficulty of the peripheral visual search task led to an increase in reaction time. In the experiments in this study, the farther the eye-controlled components were from the center of the screen, the farther the image formed on the retina was from the central concave area. According to the physiological structure of the human retina, the 1~2° field of view corresponding to the central concave area presents the clearest image, and images with low definition present at a farther position from the center of peripheral vision. In other words, the closer the eye-controlled interactive component is to the center or the larger the size, the clearer the image formed on the human retina, which helps people make quick recognition and judgment (i.e., reduce the difficulty of the search task); thus, the reaction time is decreased, which is consistent with our experimental results.

The significant interaction effect of the distance × size suggested that the size and position of eye-controlled components in an eye-controlled interface helped the users recognize objects efficiently, but when there were multiple components in the interface, they influenced each other. Objects in peripheral vision become difficult to recognize when they are in close proximity to other objects [73,74], so it is important to design eye-controlled GUI components at an appropriate distance from each other. We identified this problem in our previous study and provided relevant design recommendations [68].

In addition, the results showed that the larger the size of the components, the shorter the movement time of eye-controlled triggering; however, the distance had no significant effect on the triggering process, which was not in line with our second hypothesis (**H2**). In the experiment, participants successively completed the visual-searching and eye-controlled-triggering processes, during which they completed multiple saccades. The saccades followed the experimental paradigm of Fitts’ law, and related studies have shown that the eye-controlled selection task abided by Fitts’ law [57,60]. However, the findings on the distance of the components did not abide by Fitts’ law. This provides avenues for further analysis in conjunction with relevant studies. In terms of the mechanism of human eye movements, saccades are caused by force impulses and conform to the impulse-variance model [75]. Human eye’s natural saccades within 15° are considered small saccades, which account for 80% of the natural saccades of the human eye [76,77]. In our experiments, the distance was divided into three levels of 5.966°, 8.609°, and 11.243°, all of which were in the range of small saccades. Therefore, the lack of conformance of the experimental results with Fitts’ law is likely to be caused by small saccades. In other words, our experiments demonstrated that the amplitudes of saccades within 12° had little effect on the interaction performance in eye-controlled interaction.

Although the average reaction time for searching visual objects with abstract shapes was approximately 200 ms higher than that of concrete shapes, the ANOVA results showed that shape did not have a significant effect on movement time, which supports the third hypothesis (**H3**). Wu et al. argued that people would have a higher search efficiency for icons conforming to the semantic cognitive law [78]. Participants in our experiment performed a visual search in low-resolution images of peripheral vision and could only rely on the shape outline of the icons for recognition and judgment. In this case, abstract icons containing complex metaphors would be more difficult to recognize because concrete icons conform more to the human semantic cognitive law than abstract icons, which causes higher search efficiency. In the eye-controlled-triggering process, because the participants have already determined the direction of the interactive target, they only need to complete the saccade in the corresponding direction, and the movement process is influenced by shape factors.

The significant interaction effect of shape × distance (Figure 11e) suggests that the reaction time of abstract-shaped icons increases with the distance to the center of the visual object, while the reaction time of concrete-shaped icons does not change significantly. In other words, choosing abstract icons for eye-controlled interactive components leads to a lower recognition efficiency for users farther away from the center of the interface. Therefore, the design of eye-controlled GUI components should consider the metaphor of icons in different regions, and components far from the center of the screen should use concrete icons as much as possible to ensure that users can recognize the components efficiently. The results of the immersive subjective evaluations (Figure 13) showed that the task using abstract-shaped components in the experiment was more difficult than that using concrete-shaped components. Combined with the results of the participants’ interviews, most participants indicated that they preferred recognizing concrete-shaped components when interacting using eye control because the concrete shapes were more consistent with their expected imagination of a text prompt than the abstract shapes, which made it easier to make choices. Therefore, the use of components with concrete shapes was more consistent with the ‘user-centered’ design principle when designing eye-controlled GUI components, both from the perspective of experimental performance and user preferences.

## 5. Conclusions

Based on the results of this study and existing studies, the following recommendations for designing eye-controlled GUI components were derived:

(1) The size of the GUI elements in the eye-controlled interaction interface is very important. An appropriate size would benefit both visual-searching and eye-controlled-triggering processes. Combining the results of the subjective evaluation, we derived the recommended values of 2.889°, 3.389°, and 3.889° for size.
(2) Multiple interface components influence each other when they are in close proximity, so they need to be spaced appropriately from each other. According to our previous study [68], 1.032° is a suitable spacing value.
(3) For eye-controlled modalities that require saccades to complete triggering (e.g., eye-gesture interaction), the distance factor has less impact on the performance of eye-controlled triggering when performing saccades within 12°. In addition, if an efficient visual search is to be ensured, the recommended values for the distance of the saccades are 5.966° and 8.609°.
(4) The shape of the eye-controlled GUI components significantly affects the user recognition procedure. Abstract-shaped components can lead to a greater cognitive load than concrete-shaped components in the process of user recognition of eye-controlled interactive components. Designers should attempt to simplify the recognition process by selecting materials that fit the user’s semantic perception (e.g., using simple concrete icons).

Our research provides quantitative and standardized design recommendations for user interface design in eye-controlled interactive applications, and these findings will help improve the user experience of eye-controlled interactive applications and achieve user-centered design guidelines. For special populations with motor impairments (e.g., people with physical disabilities or ALS), using eye-controlled applications is the only way to communicate with the world. Thus, we are devoted to conducting further research focusing on the usability of eye-controlled applications, providing directions for designing human–computer interaction, and expanding the application fields of eye-controlled interaction.

## 6. Study Limitation and Future Work

This study has some limitations. In our experimental design, the values of the distance factor were determined based on three premises: (1) the participants were 64 cm away from the screen; (2) the experiment used a display with a resolution of 1920 px × 1080 px.; and (3) the experiment used a circular distribution, and the icon size was a minimum of 80 px and a maximum of 160 px. The limitation of these three premises led to a maximum value of 11.243° (less than 12°) for the distance factor, which falls within the range of values for short saccades [77]. In this case, we could not explore the situation with long saccades. Therefore, in future studies, we aim to fully consider the value of the distance factor and investigate the effect of the distance factor under long saccades.

In addition, we use Bertin’s ‘visual variables’ as the basis for determining factors [44], which consist of six factors: shape, position, area, brightness, color, and texture. We studied the first three factors through ergonomic experiments, but the last three factors also need to be considered in the GUI design for eye-controlled interaction. Therefore, we hope to investigate the other three elements in our future research.

## Figures and Tables

**Table 1:** The cognitive characteristics score of concrete-shape group and abstract-shape group.

Icons		Visual Complexity	Familiarity	Image Agreement	Name Agreement
Concrete-shape group	Mean	2.05	4.1	3.78	3.92
	N	15	15	15	15
	St. Deviation	0.41	0.55	0.5	0.59
Abstract-shape group	Mean	1.98	4.26	3.05	3.6
	N	15	15	15	15
	St. Deviation	0.26	0.41	0.42	0.38

**Table 2:** One-way Welch’s ANOVA results for reaction times of size, distance, and shape factors.

		Size					Distance			Shape	
		80 px	100 px	120 px	140 px	160 px	243 px	351 px	459 px	Abstract	Concrete
Descriptive statistics	N	120	120	120	120	120	200	200	200	300	300
	Mean	1.461	1.276	1.293	1.334	1.304	1.306	1.291	1.404	1.422	1.245
	St. Deviation	0.437	0.354	0.379	0.414	0.374	0.378	0.365	0.437	0.407	0.366
Variances homogeneity test	Levene statistic	1.776					2.136			5.097	
	df1	4					2			1	
	df2	595					597			598	
	Sig.	0.132					0.119			0.024	
Welch	Statistic	3.771					4.329			31.043	
	df1	4					2			1	
	df2	297.095					395.822			591.364	
	Sig.	0.005					0.014			0.000	

**Table 3:** One-way Welch’s ANOVA results for movement times of size, distance, and shape factors.

		Size					Distance			Shape	
		80 px	100 px	120 px	140 px	160 px	243 px	351 px	459 px	Abstract	Concrete
Descriptive statistics	N	119	119	118	119	117	198	196	198	296	296
	Mean	0.448	0.463	0.430	0.452	0.428	0.436	0.444	0.453	0.444	0.445
	St. Deviation	0.103	0.110	0.091	0.105	0.076	0.103	0.088	0.102	0.100	0.096
Variances homogeneity test	Levene statistic	2.684					2.348			0.190	
	df1	4					2			1	
	df2	587					589			590	
	Sig.	0.031					0.096			0.663	
Welch	Statistic	2.866					1.349			0.053	
	df1	4					2			1	
	df2	292.339					390.868			589.100	
	Sig.	0.024					0.261			0.818	

## Data Availability

The data presented in this study are available on request from the corresponding author.

## References

[1] Almansouri A.S. (2022). Tracking eye movement using a composite magnet. IEEE Trans. Magn..

[2] Bulling A., Roggen D., Tröster G. Wearable EOG goggles: Eye-based interaction in everyday environments. Proceedings of the 27th International Conference on Human Factors in Computing Systems, CHI 2009.

[3] Mollenbach E., Hansen J.P., Lillholm M. (2013). Eye Movements in Gaze Interaction. J. Eye Mov. Res..

[4] McSorley E. (2010). Looking and acting: Vision and eye movements in natural behaviour. Perception.

[5] Foulds R.A., Fincke R. A computerized line of gaze system for rapid non-vocal individuals. Proceedings of the National Computer Conference 1979 Personal Computing Proceedings.

[6] Rinard G., Rugg D. An ocular control device for use by the severely handicapped. Proceedings of the 1986 Conference on Systems and Devices for the Disabled.

[7] Sesin A., Adjouadi M., Cabrerizo M., Ayala M., Barreto A. (2008). Adaptive eye-gaze tracking using neural-network-based user profiles to assist people with motor disability. J. Rehabil. Res. Dev..

[8] Riman C.F. (2020). Implementation of a Low Cost Hands Free Wheelchair Controller. Indep. J. Manag. Prod..

[9] Zhang X., Kulkarni H., Morris M.R. Smartphone-Based Gaze Gesture Communication for People with Motor Disabilities. Proceedings of the 2017 CHI Conference on Human Factors in Computing Systems.

[10] Tuisku O., Majaranta P., Isokoski P., Räihä K.-J. Now Dasher! Dash away! longitudinal study of fast text entry by Eye Gaze. Proceedings of the 2008 Symposium on Eye Tracking Research & Applications.

[11] Wobbrock J.O., Robinstein J., Sawyer M.W., Duchowski A.T. Longitudinal Evaluation of Discrete Consecutive Gaze Gestures for Text Entry. Proceedings of the Eye Tracking Research and Applications Symposium.

[12] Fujii K., Salerno A., Sriskandarajah K., Kwok K.-W., Shetty K., Yang G.-Z. Gaze Contingent Cartesian Control of a Robotic Arm for Laparoscopic Surgery. Proceedings of the IEEE/RSJ International Conference on Intelligent Robots and Systems (IROS).

[13] Fujii K., Gras G., Salerno A., Yang G.-Z. (2018). Gaze gesture based human robot interaction for laparoscopic surgery. Med. Image Anal..

[14] Howell I., Vickers S., Hyrskykari A. Gaze-based interaction with massively multiplayer on-line games. Proceedings of the Conference on Human Factors in Computing Systems.

[15] Heikkilä H. EyeSketch: A Drawing Application for Gaze Control. Proceedings of the Conference on Eye Tracking South Africa.

[16] Rajanna V., Malla A.H., Bhagat R.A., Hammond T. DyGazePass: A gaze gesture-based dynamic authentication system to counter shoulder surfing and video analysis attacks. Proceedings of the IEEE International Conference on Identity.

[17] Land M.F., Hayhoe M. (2001). In what ways do eye movements contribute to everyday activities?. Vis. Res..

[18] Jacob R.J.K. (1995). Eye Tracking in Advanced Interface Design. Advanced Interface Design & Virtual Environments.

[19] Drewes H., Schmidt A. (2007). Interacting with the Computer Using Gaze Gestures. Human-Computer Interaction–INTERACT 2007.

[20] Isokoski P. Text input methods for eye trackers using off-screen targets. Proceedings of the 2000 symposium on Eye tracking research & applications.

[21] Huckauf A., Urbina M.H. (2011). Object selection in gaze controlled systems: What you don’t look at is what you get. ACM Trans. Appl. Percept..

[22] Jenke M., Huppenbauer L., Maier T. (2018). Investigation of continual operation procedures via a user centered gaze control by means of flexible gaze gestures. Z. Für Arb..

[23] Jenke M., Maier T. (2018). What Does the Eye Want? An Investigation of Interface Parameters to Ensure Intuitive Gaze-Controlled Interactions for Multidimensional Inputs.

[24] Istance H., Hyrskykari A.I. Supporting Making Fixations and the Effect on Gaze Gesture Performance. Proceedings of the 2017 CHI Conference on Human Factors in Computing Systems.

[25] Porta M., Turina M. Eye-S: A Full-Screen Input Modality for Pure Eye-based Communication. Proceedings of the 2008 Symposium on Eye Tracking Research & Applications.

[26] Urbina M.H., Huckauf A. Alternatives to single character entry and dwell time selection on eye typing. Proceedings of the 2010 Symposium on Eye-Tracking Research & Applications.

[27] Bee N., André E. Writing with Your Eye: A Dwell Time Free Writing System Adapted to the Nature of Human Eye Gaze. Proceedings of the 4th IEEE tutorial and research workshop on Perception and Interactive Technologies for Speech-Based Systems.

[28] Jungwirth F., Haslgrübler M., Ferscha A. Contour-guided gaze gestures: Using object contours as visual guidance for triggering interactions. Proceedings of the 2018 ACM Symposium on Eye Tracking Research & Applications.

[29] Niu Y., Li X., Yang W., Xue C., Peng N., Jin T. (2021). Smooth Pursuit Study on an Eye-Control System for Continuous Variable Adjustment Tasks. Int. J. Hum. Comput. Interact..

[30] Hou W.-J., Wu S.-Q., Chen X.-L., Chen K.-X. (2019). Study on Spatiotemporal Characteristics of Gaze Gesture Input. Human-Computer Interaction. Recognition and Interaction Technologies.

[31] Menges R., Kumar C., Müller D., Sengupta K. GazeTheWeb: A Gaze-Controlled Web Browser. Proceedings of the 14th International Web for All Conference.

[32] Porta M., Ravelli A. WeyeB, an eye-controlled Web browser for hands-free navigation. Proceedings of the 2009 2nd Conference on Human System Interactions.

[33] Menges R., Kumar C., Sengupta K., Staab S. eyeGUI: A Novel Framework for Eye-Controlled User Interfaces. Proceedings of the 9th Nordic Conference on Human-Computer Interaction (NordiCHI).

[34] Desimone R. (1995). Neural Mechanisms of Selective Visual Attention. Annu. Rev. Neurosci..

[35] Neisser U. (2014). Focal Attention and Figural Synthesis. Cognitive Psychology.

[36] Bundesen C. (1990). A theory of visual attention. Psychol. Rev..

[37] Trevarthen C.B. (1968). Two mechanisms of vision in primates. Psychol. Forsch..

[38] Meng X., Wang Z. A pre-attentive model of biological vision. Proceedings of the 2009 IEEE International Conference on Intelligent Computing and Intelligent Systems.

[39] Paillard J., Amblard B. (1985). Static versus Kinetic Visual Cues for the Processing of Spatial Relationships. Brain Mechanisms and Spatial Vision.

[40] Khan M., Lawrence G., Franks I., Buckolz E. (2004). The utilization of visual feedback from peripheral and central vision in the control of direction. Exp. Brain Res..

[41] Roux-Sibilon A., Trouilloud A., Kauffmann L., Guyader N., Mermillod M., Peyrin C. (2019). Influence of peripheral vision on object categorization in central vision. J. Vis..

[42] Sharkey T.J., Hennessy R.T. Head-mounted, ambient vision display for helicopter pilotage. Proceedings of the Conference on Helmet- and Head-Mounted Displays V.

[43] Lenneman J.K., Backs R.W. (2018). A psychophysiological and driving performance evaluation of focal and ambient visual processing demands in simulated driving. Transp. Res. Part F-Traffic Psychol. Behav..

[44] Barbut M. (1967). Sémiologie Graphique.

[45] Niu Y.F., Liu J., Cui J.Q., Yang W.J., Zuo H.R., He J.X., Xiao L., Wang J.H., Ma G.R., Han Z.J. (2022). Research on visual representation of icon colour in eye-controlled systems. Adv. Eng. Inform..

[46] Weelden L.V., Cozijn R., Maes A., Schilperoord J. Perceptual similarity in visual metaphor processing. Proceedings of the Cognitive Shape Processing, Papers from the 2010 AAAI Spring Symposium, Technical Report SS-10-02.

[47] Jansen B.J. (1998). The Graphical User Interface. Acm Sigchi Bull..

[48] Boyd L.H., Others A. (1990). The Graphical User Interface: Crisis, Danger, and Opportunity. J. Vis. Impair. Blind..

[49] Humphreys G.W., Forde E.M.E. (2001). Hierarchies, similarity, and interactivity in object recognition: “Category-specific” neuropsychological deficits. Behav. Brain Sci..

[50] Biederman I. (1987). Recognition-by-components: A theory of human image understanding. Psychol. Rev..

[51] Van Weelden L., Maes A., Schilperoord J., Cozijn R. (2011). The Role of Shape in Comparing Objects: How Perceptual Similarity May Affect Visual Metaphor Processing. Metaphor. Symb..

[52] Rosch E., Mervis C.B., Gray W.D., Johnson D.M., Boyes-Braem P. (1976). Basic objects in natural categories. Cogn. Psychol..

[53] Aldrich V.C. (1968). Visual metaphor. J. Aesthetic Educ..

[54] Carroll N. (1994). Visual metaphor. Aspects of Metaphor.

[55] Kumar M. (2007). Gaze-Enhanced User Interface Design. Ph.D. Thesis.

[56] Fitts P.M. (1954). The information capacity of the HUMAN motor system In Controlling the Amplitude of movement. J. Exp. Psychol..

[57] Ware C., Mikaelian H.H. An evaluation of an eye tracker as a device for computer input2. Proceedings of the SIGCHI/GI Conference on Human Factors in Computing Systems and Graphics Interface.

[58] Miniotas D. Application of Fitts’ law to eye gaze interaction. Proceedings of the CHI00: Conference on Human Factors In Computing systems.

[59] Mackenzie I.S. (1992). Fitts’ Law as a Research and Design Tool in Human-Computer Interaction. Hum. Comput. Interact..

[60] Vertegaal R. A Fitts Law comparison of eye tracking and manual input in the selection of visual targets. Proceedings of the 10th International Conference on Multimodal Interfaces, ICMI 2008.

[61] Snodgrass J.G., Vanderwart M. (1980). A standardized set of 260 pictures: Norms for name agreement, image agreement, familiarity, and visual complexity. J. Exp. Psychol. Hum. Learn..

[62] Georgopoulos A. (1990). Neural coding of the direction of reaching and a comparison with saccadic eye movements. Cold Spring Harb. Symp. Quant. Biol..

[63] Faul F., Erdfelder E., Lang A.-G., Buchner A. (2007). G*Power 3: A flexible statistical power analysis program for the social, be-havioral, and biomedical sciences. Behav. Res. Methods.

[64] Land M.F., Furneaux S. (1997). The knowledge base of the oculomotor system. Philos. Trans. R. Soc. Lond. Ser. B Biol. Sci..

[65] Jacob R.J.K. (1991). The use of eye movements in human-computer interaction techniques: What you look at is what you get. ACM Trans. Inf. Syst..

[66] Ovarfordt P. Conversing with the user based on eye-gaze patterns. Proceedings of the SIGCHI Conference on Human Factors in Computing Systems.

[67] Majaranta P., Mackenzie I.S., Aula A., Räihä K.-J. (2006). Effects of feedback and dwell time on eye typing speed and accuracy. Univers. Access Inf. Soc..

[68] Niu Y.F., Zuo H.R., Yang X., Xue C.Q., Peng N.Y., Zhou L., Zhou X.Z., Jin T. (2021). Improving accuracy of gaze-control tools: Design recommendations for optimum position, sizes, and spacing of interactive objects. Hum. Factors Ergon. Manuf. Serv. Ind..

[69] Field A. (2005). Discovering statistics using R. Discovering Statistics Using SPSS.

[70] Treisman A., Gormican S. (1988). Feature analysis in early vision: Evidence from search asymmetries. Psychol. Rev..

[71] Zelinsky G., Sheinberg D. (1995). Why some search tasks take longer than others: Using eye movements to redefine reaction times. studies in visual information processing. Stud. Vis. Inf. Process..

[72] Hooge I., Erkelens C.J. (1999). Peripheral vision and oculomotor control during visual search. Vis. Res..

[73] Pelli D.G. (2008). Crowding: A cortical constraint on object recognition. Curr. Opin. Neurobiol..

[74] Whitney D., Levi D.M. (2011). Visual crowding: A fundamental limit on conscious perception and object recognition. Trends Cogn..

[75] Abrams R.A., Meyer D.E., Kornblum S. (1989). Speed and Accuracy of Saccadic Eye Movements: Characteristics of Impulse Variability in the Oculomotor System. J. Exp. Psychol. Hum. Percept. Perform..

[76] Bahill A.T., Adler D., Stark L. (1975). Most naturally occurring human saccades have magnitudes of 15 degrees or less. Investig. Ophthalmol..

[77] Bahill A.T., Stark L. (1975). Overlapping saccades and glissades are produced by fatigue in the saccadic eye movement system. Exp. Neurol..

[78] Wu X., Yan H., Niu J., Gu Z. (2022). Study on semantic-entity relevance of industrial icons and generation of metaphor design. J. Soc. Inf. Display.

